# Multivalvular Carcinoid Heart Disease: The Role of Echocardiography in Diagnosis and Selection for Heterotopic Bicaval Valve Implantation

**DOI:** 10.3390/diagnostics16121942

**Published:** 2026-06-22

**Authors:** Bianca Corrêa Rocha de Mello, Ana Clara Pierote Rodrigues Vasconcelos, Mariana Ubaldo Barbosa Paiva, Mateus Veloso e Silva, Nattália de Oliveira Maciel, Priscila Ribeiro de Andrade, Rodolfo Deusdará, Maria Estefânia Bosco Otto

**Affiliations:** 1 University of Brasilia Medical School, Brasilia 70910-900, DF, Brazil; biancacorrea@gmail.com (B.C.R.d.M.); anavasconcelos2002@gmail.com (A.C.P.R.V.); mubp2505@gmail.com (M.U.B.P.); nattaliamaciel888@gmail.com (N.d.O.M.); rodolfodeusdara@gmail.com (R.D.); 2 Brazilian Company of Hospital Services, University of Brasilia (HUB-UnB/EBSERH), Brasilia 70840-901, DF, Brazil; mateusvlsilva@gmail.com (M.V.e.S.); tspriscilaandrade@gmail.com (P.R.d.A.)

**Keywords:** carcinoid heart disease, carcinoid syndrome, neuroendocrine carcinoma, heart valve disease, transcatheter procedures

## Abstract

**Background and Clinical Significance**: Carcinoid heart disease (CHD) is an uncommon valvular manifestation of neuroendocrine tumours, usually affecting right-sided cardiac valves. Left-sided involvement is rare and is generally associated with bronchopulmonary carcinoid, right-to-left shunting, or markedly elevated circulating vasoactive substances. Therapeutic decision-making is particularly challenging in advanced disease when severe tricuspid regurgitation occurs in patients at prohibitive surgical risk. **Case Presentation**: We report the case of a 61-year-old male patient with progressive dyspnoea, abdominal distension, lower-limb oedema, facial flushing, and 15 kg of unintentional weight loss. Transthoracic and transoesophageal echocardiography demonstrated torrential tricuspid regurgitation caused by thickened, retracted, and immobile leaflets, with additional mitral and aortic valve involvement, raising strong suspicion of CHD. An agitated-saline contrast study demonstrated delayed right-to-left shunting without patent foramen ovale, suggesting an extracardiac, likely intrapulmonary, shunt. Somatostatin receptor PET/CT identified a pancreatic lesion with metastatic disease, and bone marrow biopsy confirmed neuroendocrine tumour infiltration. Owing to prohibitive surgical risk, as reflected by a Tricuspid Regurgitation Impact Score (TRI-SCORE) with an estimated in-hospital mortality of 65%, unfavourable tricuspid anatomy for repair, and refractory venous congestion, heterotopic bicaval valve implantation was performed (TricValve system -P&F). **Discussion**: This case highlights the role of echocardiography in recognising the characteristic phenotype of CHD, detecting occult right-to-left shunting, and supporting selection of a palliative transcatheter intervention. It also illustrates the value of a multimodality diagnostic strategy integrating echocardiography, functional oncological imaging, and histopathology in tumour-related cardiac disease. **Conclusions**: In selected inoperable patients with advanced carcinoid-related tricuspid regurgitation, heterotopic bicaval valve implantation may represent a feasible strategy for reducing venous congestion and improving functional status.

## 1. Introduction

Neuroendocrine neoplasms are rare malignancies that most commonly arise from gastroenteropancreatic structures [1]. When well differentiated, they are classified as neuroendocrine tumours (NETs) [2]. Although uncommon, NETs have shown an increasing reported incidence in recent decades, probably due to greater clinical awareness and advances in diagnostic techniques [3,4]. A subset of functional NETs may lead to carcinoid syndrome (CS), a paraneoplastic condition caused by the systemic release of serotonin, also known as 5-hydroxytryptamine (5-HT), and other vasoactive mediators [2,5].

Carcinoid heart disease (CHD) represents one of the most clinically relevant complications of CS. Chronic exposure to serotonin and related humoral factors promotes fibroblast activation and plaque-like fibrous deposition on the endocardial surfaces of valve leaflets, the subvalvular apparatus, and cardiac chambers [6,7]. This process results in the characteristic echocardiographic phenotype of CHD, with thickened, retracted, and immobile valve leaflets, most often affecting the tricuspid and pulmonary valves. The clinical presentation may include progressive right-sided heart failure, with dyspnoea, fatigue, peripheral oedema, ascites, and hepatomegaly [5].

The left-sided cardiac valves are usually spared because vasoactive substances are generally degraded in pulmonary circulation. However, left-sided involvement has been reported in approximately 10–15% of cases worldwide, extending to 20.83% in a Latin American cohort, and is generally associated with a patent foramen ovale (PFO), pulmonary metastases, or markedly elevated circulating levels of vasoactive substances [8,9,10]. Recognising this uncommon pattern is diagnostically important, particularly because multivalvular involvement may mimic rheumatic heart disease or other fibrotic valvular disorders.

The diagnostic evaluation of CHD relies on an integrated approach combining clinical assessment, biochemical markers, echocardiography, and imaging for tumour localisation and staging [6]. Among these modalities, echocardiography has a pivotal role because it is often the first examination to suggest the diagnosis. Transthoracic echocardiography (TTE) allows the recognition of the characteristic valvular phenotype, grading of tricuspid and pulmonary valve disease, and assessment of right-sided chamber remodelling and ventricular function [11]. In atypical or left-sided involvement, transoesophageal echocardiography (TEE) may further define valve morphology and identify mechanisms that explain systemic exposure to vasoactive mediators.

For this reason, screening for CHD is recommended in patients with suspected or confirmed CS. Urinary or plasma 5-hydroxyindoleacetic acid (5-HIAA) should be measured, followed by N-terminal pro-B-type natriuretic peptide (NT-proBNP) assessment when 5-HIAA is elevated. If NT-proBNP exceeds 260 ng/mL or if the patient is symptomatic, TTE is recommended. In addition, agitated-saline contrast echocardiography may help detect right-to-left shunts, which are particularly relevant when left-sided valve involvement is present. This technique may also contribute to distinguishing intracardiac from extracardiac shunting, refining the pathophysiological interpretation of unusual left-sided CHD. Selected cases may require cardiovascular magnetic resonance, cardiac computed tomography (CT), or positron emission tomography/computed tomography (PET/CT) using radiolabelled somatostatin analogues [12,13].

Beyond diagnosis, imaging plays an essential role in therapeutic planning. Echocardiography provides information on valve anatomy, coaptation defects, right ventricular size and function [14], and the severity of systemic venous congestion, all of which are relevant when considering surgical, transcatheter, or palliative strategies [15,16]. Multimodality imaging may also help define the tumour burden, metastatic spread, and feasibility of oncological or interventional treatment [17].

The clinical course of CHD is difficult to predict. Because CS is associated with impaired quality of life, treatment strategies aim to reduce the tumour burden and suppress the secretion of biogenic amines [18,19]. Somatostatin analogues are central to symptom control, whereas surgery may be considered in selected patients who can tolerate and benefit from cytoreduction [20,21]. In advanced CHD, therapeutic decisions are complex and require a multidisciplinary heart-team approach, integrating tumour status, valve anatomy, right ventricular (RV) function, surgical risk, and feasibility of transcatheter or palliative interventions [22,23].

In this report, we describe a patient with metastatic NET and multivalvular CHD with right and left-sided involvement. Here, echocardiography played a central role in recognising the disease phenotype, identifying an extracardiac right-to-left shunt, and supporting the decision for heterotopic bicaval valve implantation as a palliative strategy.

## 2. Case Presentation

### 2.1. Clinical Data

A 61-year-old man presented with a two-year history of progressive dyspnoea, abdominal distension, lower-limb oedema, occasional facial flushing, and unintentional weight loss of 15 kg over the preceding six months. He had been treated for heart failure of undetermined aetiology, with only partial symptomatic improvement.

Physical examination revealed jugular venous distension, a holosystolic murmur at the lower left sternal border, hepatomegaly, ascites, and bilateral lower-limb oedema. At admission, he was receiving pantoprazole, spironolactone, furosemide, carvedilol, enalapril, and dapagliflozin. During hospitalisation, decongestive therapy was intensified because of persistent systemic venous congestion, with furosemide increasing to 240 mg/day. Spironolactone and hydrochlorothiazide were maintained as adjunctive diuretic therapy to enhance natriuresis in association with loop diuretic treatment. Despite this intensified regimen, the patient remained markedly congested, with refractory ascites and peripheral oedema. Owing to persistent systemic congestion, he was referred for cardiological reassessment and comprehensive imaging evaluation.

### 2.2. Echocardiographic Assessment

**Figure 1:**
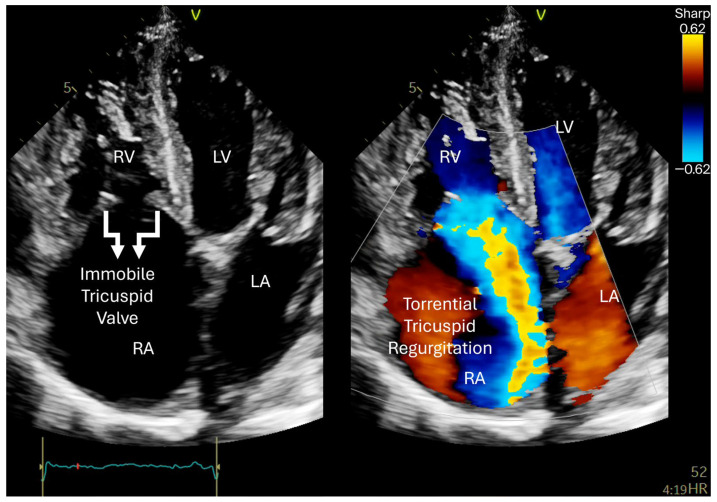
TTE. Apical four-chamber view demonstrating marked right atrium (RA) and right ventricular (RV) dilatation, with thickened, retracted, and immobile tricuspid valve leaflets, resulting in a large coaptation defect. Colour Doppler imaging shows torrential TR, consistent with advanced carcinoid-related tricuspid valve disease. LV: left ventricle; LA: left atrium. See also Video S1.

**Figure 2:**
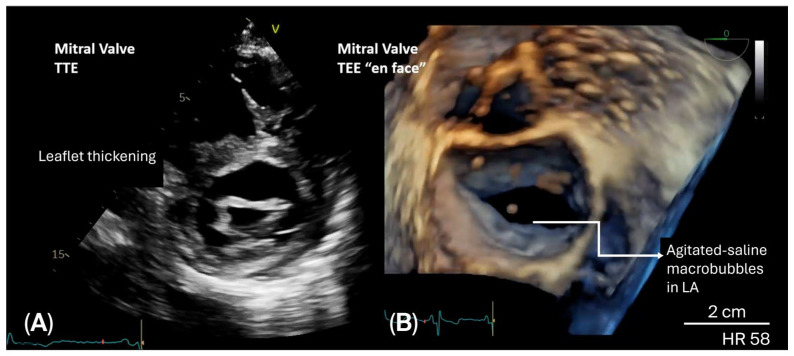
Mitral valve involvement and agitated-saline injection in TEE. (**A**) TTE showing thickening of the mitral valve leaflets, consistent with left-sided valvular involvement. (**B**) Three-dimensional TEE, with en face view of the mitral valve (MV), demonstrating agitated-saline macrobubbles in the left atrium (LA), suggesting right-to-left shunting and confirming the thickened MV.

TTE revealed torrential tricuspid regurgitation (TR) with markedly thickened, retracted, and immobile leaflets, associated with severe right chamber dilatation (Figure 1; Video S1). The mitral valve (MV) showed leaflet thickening, commissural fusion, severe regurgitation, and mild stenosis, with a planimetric area of 2.4 cm^2^ (Figure 2A). The aortic valve was thickened, with leaflet retraction and moderate regurgitation. Biventricular systolic function was mildly reduced, with a left ventricular biplane ejection fraction of 49%.

**Figure 3:**
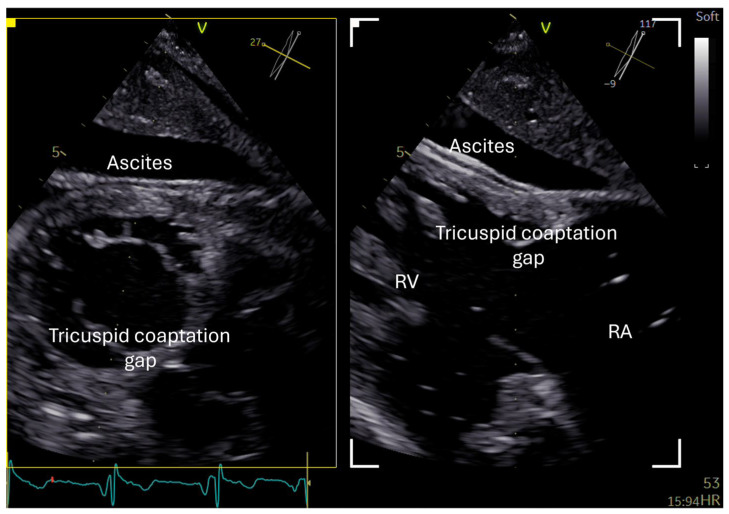
(Video S2) TEE (transgastric view). Orthogonal transgastric view of TV showed severe thickening, retraction, and immobility of valve leaflets and a large coaptation gap. Ascites is also visible, reflecting advanced systemic venous congestion. RV: right ventricle; RA: Right atrium.

**Figure 4:**
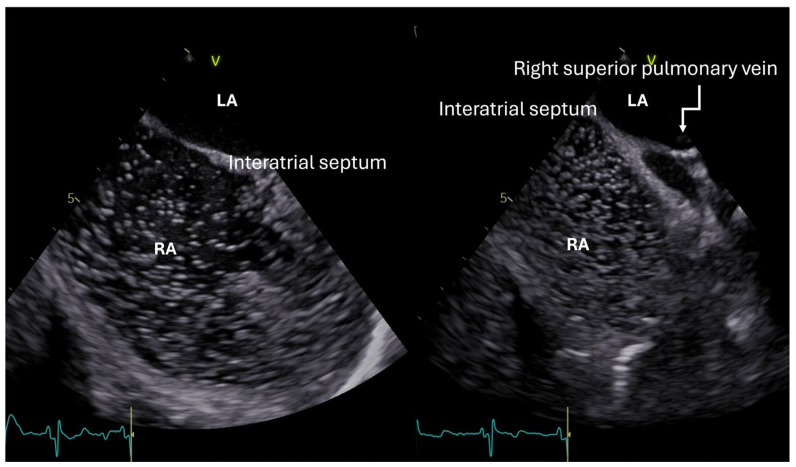
Agitated-saline infusion during TEE. Orthogonal TEE views of the interatrial septum during agitated-saline infusion show opacification of the RA, without macrobubble passage across the region of the interatrial septum. No immediate passage of bubbles across the interatrial septum was observed, including during the Valsalva manoeuvre. Delayed opacification of the LA occurred after more than five cardiac cycles, with macrobubbles visualised entering from the right superior pulmonary vein, suggesting an extracardiac, presumably intrapulmonary shunt. The dynamic sequence is shown in Supplementary Video S3.

This multivalvular pattern of leaflet thickening, retraction, restricted mobility, and predominant regurgitation raised strong suspicion of CHD. TEE confirmed the valvular abnormalities (Figure 2B, Figure 3 and Video S2). During agitated-saline contrast TEE, no immediate passage of macrobubbles across the interatrial septum was observed, even during the Valsalva manoeuvre with leftward septal bowing. Instead, macrobubbles appeared late in the LA, after more than five cardiac cycles, and were directly visualised entering from the right superior pulmonary vein, suggesting an extracardiac, presumably intrapulmonary, right-to-left shunt (Figure 4, Video S3) [24], without evidence of a PFO or atrial septal defect.

### 2.3. Biochemical and Multimodality Diagnostic Work-Up

Biochemical testing of plasma and urine samples collected under appropriate dietary restrictions showed... markedly elevated neuroendocrine tumour markers: chromogranin A, 1020 ng/mL (reference <108); serotonin, 2088.94 ng/mL (reference, 50–250); and 24 h urinary 5-HIAA, 41.4 mg (reference, 1.5–6.5).

**Figure 5:**
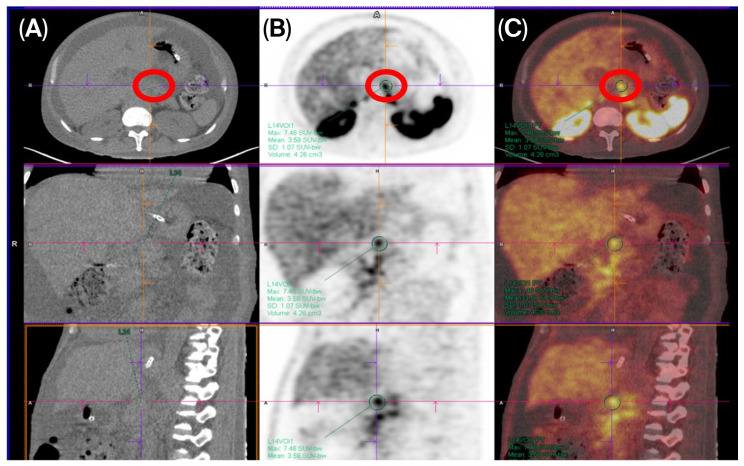
Somatostatin receptor PET/CT showing the pancreatic primary lesion. Abdominal PET/CT described a focal lesion in the pancreatic body, highlighted by the red circle. (**A**) Computed tomography image showing the anatomical location of the pancreatic lesion. (**B**) Functional PET image demonstrating increased radiotracer uptake in the corresponding region, consistent with somatostatin receptor expression. (**C**) Fused PET/CT image confirming the hypermetabolic lesion in the pancreatic body.

**Figure 6:**
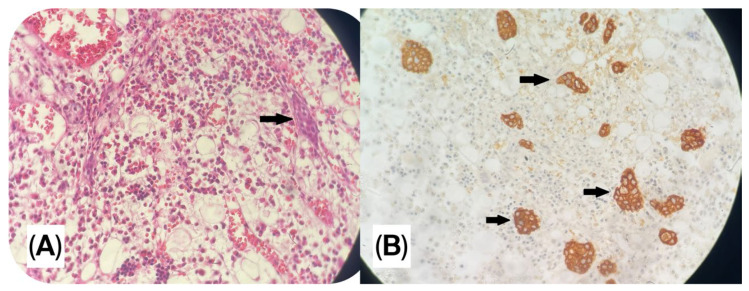
Photomicrographs of bone marrow biopsy. (**A**) Haematoxylin and eosin staining showing bone marrow infiltration by cells with NET morphology (arrow). (**B**) Immunohistochemical staining for chromogranin A demonstrates positive expression in tumour cell clusters (arrows).

The primary NET was presumed to arise from the pancreatic body, as 18F-NOTA PET/CT using a fluorine-18-labelled somatostatin analogue demonstrated a focal hypermetabolic lesion at this site (Figure 5), in association with multiple lymph node metastases and blastic bone lesions. Given the high procedural risk associated with biopsy of the pancreatic lesion, bone marrow biopsy was selected as the safest available approach for tissue diagnosis. Histopathological examination demonstrated infiltration by cells with neuroendocrine morphology, and immunohistochemistry showed chromogranin A positivity, confirming metastatic NET involvement of the bone marrow (Figure 6). Abdominal magnetic resonance imaging was non-diagnostic for the primary lesion.

### 2.4. Diagnosis and Heart Team Decision

Following integrated assessment by the institutional multidisciplinary Clinical and Heart Team at University Hospital, including specialists from Cardiology, Cardiovascular Imaging, Interventional Cardiology, Oncology, Nuclear Medicine, and Pathology, the final diagnosis was established as multivalvular CHD secondary to metastatic NET, with rare left-sided involvement attributed to an extracardiac right-to-left shunt. The patient’s surgical risk was objectively assessed using the official web-based Tricuspid Regurgitation Impact Score (TRI-SCORE) calculator for isolated tricuspid valve surgery (https://www.tri-score.com/). The URL was accessed on 19 June 2026. The TRI-SCORE model incorporates eight preoperative clinical, laboratory, and echocardiographic variables: age ≥70 years, NYHA functional class III–IV, signs of right-sided heart failure, daily furosemide dose ≥125 mg, glomerular filtration rate <30 mL/min, elevated total bilirubin level, left ventricular ejection fraction (LVEF) <60%, and moderate or severe right ventricular (RV) dysfunction. Although the model includes eight variables, the final score ranges from 0 to 12 points because some variables are weighted more heavily. A TRI-SCORE of ≤3 is generally considered to indicate low surgical risk, whereas scores of 4–5 and ≥6 correspond to intermediate and high surgical risk, respectively. The calculated TRI-SCORE of this patient was 10/12, corresponding to a predicted in-hospital mortality of 65%. The positive variables included NYHA functional class III/IV, signs of right-sided heart failure, high-dose loop diuretic requirement during hospitalisation, glomerular filtration rate <30 mL/min, elevated total bilirubin, left ventricular ejection fraction <60%. This high-risk profile, together with advanced metastatic disease and unfavourable tricuspid valve anatomy for transcatheter edge-to-edge repair, supported the Heart Team decision to avoid conventional surgery and select heterotopic bicaval valve implantation as a palliative strategy [25]. Transcatheter edge-to-edge repair was also considered unsuitable because of markedly restricted and almost immobile tricuspid leaflets, a large coaptation gap of 22 mm, and severe annular dilatation, with a tricuspid annular diameter of 60 mm [22].

Given persistent systemic venous congestion despite medical therapy, the patient was selected for heterotopic bicaval valve implantation using the TricValve system (P&F).

### 2.5. Intervention and Follow-Up

**Figure 7:**
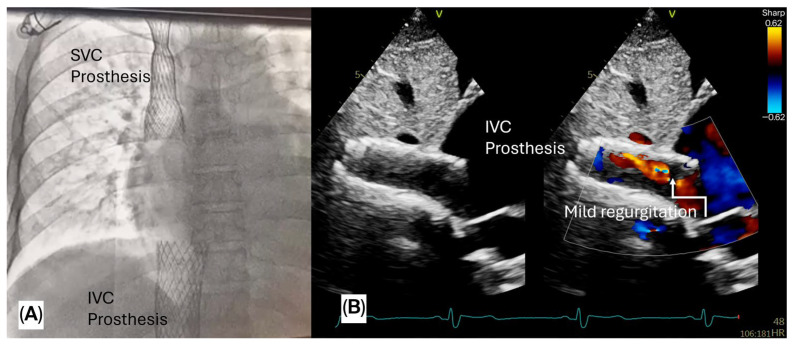
Heterotopic bicaval valve implantation using the TricValve system. (**A**) Fluoroscopy showing the final position of the prostheses in the superior (SVC) and inferior vena cavae (IVC). (**B**) Post-implant echocardiographic image from the subcostal view of the IVC prosthesis, with colour Doppler demonstrating mild residual regurgitation.

Heterotopic bicaval valve implantation using the TricValve system (P&F) was successfully performed (Figure 7). The procedure was uneventful, with no immediate complications. This palliative intervention was chosen to reduce caval backflow, alleviate systemic venous congestion, and achieve haemodynamic stabilisation before the initiation of oncological treatment [26].

The main pre- and post-procedural echocardiographic findings are summarised in Table 1. At the early post-procedural assessment, the native tricuspid valve lesion remained unchanged, as expected after heterotopic bicaval valve implantation. However, the follow-up echocardiogram showed improved caval/hepatic venous reflux patterns, preserved RV systolic function despite persistent right ventricular enlargement, and a reduction in MR severity from severe to moderate.

During follow-up, the patient showed marked clinical improvement, with better control of systemic venous congestion under optimised medical therapy. Functional status improved from NYHA functional class IV before the intervention to NYHA class II at 6-month follow-up after heterotopic bicaval valve implantation. The 6 min walk distance increased from 337 m at the first post-procedural assessment to 397 m at 6-month follow-up, corresponding to an absolute improvement of 60 m. This clinical and functional improvement was consistent with the benefits reported after TricValve implantation in high-risk patients with severe tricuspid regurgitation [26]. The patient remained under multidisciplinary follow-up by the cardiology and oncology teams.

## 3. Discussion

### 3.1. Diagnostic Relevance of the Present Case

The present case illustrates a rare and diagnostically challenging presentation of CHD, characterised by extensive multivalvular involvement, including left-sided valve disease, in a patient initially treated for heart failure of undetermined aetiology. The diagnosis was not suspected on clinical grounds alone, but was raised by recognition of a distinctive echocardiographic phenotype.

The most relevant finding was torrential TR caused by markedly thickened, retracted, and immobile leaflets, associated with severe right-sided chamber dilatation. This morphology reflects the typical fibrotic valvular involvement described in CHD, in which plaque-like deposition affects valve leaflets, the subvalvular apparatus, and endocardial surfaces [6,7]. In this patient, concomitant mitral and aortic valve abnormalities increased the diagnostic complexity, because some mitral features partially overlapped with rheumatic valve disease. However, the predominance of severe right-sided involvement, the multivalvular regurgitant pattern, and the subsequent biochemical and oncological findings supported CHD as the unifying diagnosis.

### 3.2. Echocardiography and Shunt Detection

Beyond the initial diagnostic suspicion, TEE was essential for clarifying the mechanism of left-sided involvement [7]. In the present case, agitated-saline contrast TEE showed no immediate interatrial passage of macrobubbles, even during the Valsalva manoeuvre. Instead, macrobubbles appeared late in the LA, after more than five cardiac cycles, and were visualised entering through the right superior pulmonary vein, suggesting an extracardiac, presumably intrapulmonary, right-to-left shunt (Video S3). Several mechanisms may account for intrapulmonary shunting in this setting, including microscopic pulmonary vascular dilatation, pulmonary metastatic involvement with abnormal vascular communications, or less commonly, a structural pulmonary arteriovenous malformation. Although no PFO or atrial septal defect was identified, the presumed intrapulmonary origin of the shunt was inferred from the delayed timing of bubble appearance and its visualisation entering the LA from a pulmonary vein during TEE.

Left-sided valvular involvement is uncommon in CHD because vasoactive substances are usually degraded in the pulmonary circulation before reaching the left-sided cardiac valves. Reported mechanisms include bronchopulmonary carcinoid, intracardiac right-to-left shunting, pulmonary metastases or markedly elevated circulating levels of vasoactive substances, as described by Igawa et al. [8,9,27]. In the present case, the identification of a probable extracardiac shunt provided a plausible mechanism for systemic exposure of the mitral and aortic valves to serotonin and other mediators. Therefore, agitated-saline contrast echocardiography should be considered in patients with suspected CHD and atypical left-sided valve lesions, particularly when conventional imaging does not identify an intracardiac communication [12].

### 3.3. Contribution of Multimodality Imaging and Histology

Although echocardiography was decisive in raising suspicion of CHD, confirmation of the underlying aetiology required integration with biochemical testing, functional tumour imaging, and histology. Markedly elevated chromogranin A, serotonin, and 24 h urinary 5-HIAA levels supported the diagnosis of carcinoid syndrome, as these biomarkers reflect neuroendocrine tumour activity and serotonin metabolism [5,7].

Somatostatin receptor PET/CT then identified the probable primary pancreatic lesion and demonstrated metastatic disease, including lymph node and bone involvement. This finding was particularly relevant because abdominal magnetic resonance imaging was non-diagnostic for the primary site, reinforcing the incremental value of functional imaging in tumour localisation and staging [18]. Bone marrow biopsy with immunohistochemistry confirmed infiltration by neuroendocrine tumour cells. However, bone marrow infiltration by NET cells is rarely reported in CS and usually reflects advanced disseminated disease. Özkan et al. described a similarly uncommon case of rectal carcinoid tumour with CS, osteoblastic bone metastases, and bone marrow involvement confirmed by biopsy., highlighting the diagnostic relevance of marrow evaluation in selected patients with extensive metastatic NET [28]. Thus, the final diagnosis resulted from a stepwise diagnostic pathway: echocardiographic suspicion, biochemical confirmation of hormonal activity, PET/CT-based tumour localisation and staging, and histological confirmation of metastatic NET.

This sequential strategy illustrates how echocardiography, nuclear imaging, biochemical assessment, and histopathology can provide complementary information in patients with suspected tumour-related cardiac disease.

### 3.4. Therapeutic Decision-Making and Surgical Risk

The therapeutic strategy in this case was determined by a multidisciplinary heart team, considering the severity of systemic venous congestion, valve anatomy, right ventricular involvement, tumour burden, and predicted surgical risk. This approach is consistent with the need for individualised management in CHD, integrating oncological status and cardiovascular disease severity [18,22].

Conventional tricuspid valve surgery was not considered appropriate because the patient had advanced metastatic disease, refractory venous congestion, severe tricuspid leaflet restriction, annular dilatation, and very high predicted perioperative risk. The TRI-SCORE was particularly important in this decision. The patient had a calculated TRI-SCORE of 10/12, corresponding to an estimated in-hospital mortality of 65% [25]. This objective risk assessment supported the classification of the patient as having prohibitive surgical risk, rather than simply being a high-risk surgical candidate.

In this context, conventional surgery would have been clinically disproportionate, especially considering the palliative goals of care and the need to stabilise the patient before oncological treatment. Transcatheter edge-to-edge repair was also considered unsuitable. The markedly thickened, retracted, and immobile tricuspid leaflets, together with the large coaptation defect, made adequate leaflet grasping unlikely [22]. These anatomical features are common in advanced carcinoid-related tricuspid valve disease and represent an important limitation for repair-based transcatheter strategies [6,7].

### 3.5. Rationale for Heterotopic Bicaval Valve Implantation

Heterotopic bicaval valve implantation using the TricValve system (P&F) was selected as a palliative haemodynamic strategy. Unlike surgical replacement or orthotopic transcatheter tricuspid valve replacement, this approach does not directly treat the diseased tricuspid valve. Instead, it aims to reduce systolic backflow into the superior and inferior vena cava, thereby attenuating systemic venous congestion [22,29].

This distinction is important in the present case. The clinical burden was dominated by refractory ascites, hepatomegaly, peripheral oedema, and persistent congestion despite optimised medical therapy. Therefore, the goal of the intervention was not anatomical correction of the tricuspid valve, but symptomatic and haemodynamic improvement. Heterotopic bicaval valve implantation was considered suitable because it could reduce caval reflux without requiring manipulation of the severely fibrotic and immobile tricuspid leaflets [29].

Heterotopic bicaval valve implantation in CHD has previously been reported by Stolz et al. [30]. However, that case did not involve left-sided valvular disease. Therefore, to the best of our knowledge, the present report appears to be the first description of TricValve implantation in a patient with multivalvular carcinoid involvement of left-sided valves. This approach is particularly relevant in carcinoid heart disease, in which leaflet fibrosis, retraction, and immobility often limit conventional reparative strategies [6,7,22].

### 3.6. Clinical Implications

This case emphasises the importance of maintaining a high index of suspicion for CHD when echocardiography demonstrates a characteristic pattern of right-sided valvular fibrosis and severe regurgitation. Left-sided involvement should prompt systematic evaluation for right-to-left shunting and for mechanisms that allow systemic exposure to vasoactive mediators [8,9,12].

The case further supports the value of a multimodality diagnostic strategy in cardio-oncology. Each modality provided complementary information rather than duplicating findings: TTE identified the haemodynamic consequences of valvular disease and raised suspicion for CHD; TEE refined anatomical assessment, excluded an intracardiac shunt, and suggested an extracardiac right-to-left shunt; PET/CT established tumour localisation and metastatic spread; and histology confirmed the diagnosis [7,12]. This integrated approach is consistent with recent evidence on suspected tumour-related cardiac disease, in which echocardiography is proposed as the first-line examination, while CT, cardiac magnetic resonance, and PET provide incremental anatomical, tissue characterisation, and metabolic information according to the clinical scenario [31]. In addition, objective surgical risk assessment using TRI-SCORE contributed to Heart Team decision-making in a patient with prohibitive operative risk [25].

This case also has important limitations. As a single-case report, it does not allow conclusions regarding the generalisability, durability, or prognostic impact of heterotopic bicaval valve implantation in CHD. In addition, although saline contrast TEE strongly suggested an extracardiac, presumably intrapulmonary, shunt because of delayed bubble appearance and direct visualisation of bubbles entering through the right superior pulmonary vein, no dedicated anatomical imaging was performed to definitively exclude a structural pulmonary arteriovenous malformation. Therefore, the mechanism of the extracardiac shunt should be interpreted with caution. Moreover, the pancreatic lesion was not directly biopsied because of procedural risk, and the final diagnosis was based on integrated multimodality imaging, biochemical, histopathological, and echocardiographic findings. Nevertheless, the clinical improvement observed after the procedure suggests that this approach may be considered in carefully selected inoperable patients with advanced carcinoid-related TR. Prospective registries and multicentre studies are needed to clarify patient selection, timing of intervention, haemodynamic durability, interaction with oncological therapy, and long-term clinical outcomes.

In summary, the present case demonstrates how echocardiography can move beyond diagnosis and directly influence therapeutic decision-making in advanced CHD. In patients with severe carcinoid-related TR, refractory venous congestion, unsuitable valve anatomy for repair, and prohibitive surgical risk, a palliative transcatheter strategy may improve haemodynamic status and facilitate subsequent oncological management [18,22].

## 4. Conclusions

This case illustrates the characteristic valvular phenotype of CHD and highlights the central role of echocardiography in recognising this rare but distinctive entity. The combination of leaflet thickening, retraction, and restricted mobility, particularly when involving the tricuspid valve, should prompt suspicion of CHD in patients with right-sided heart failure and systemic venous congestion. Agitated-saline contrast TEE may help identify right-to-left shunting when left-sided valve involvement is present and no PFO is detected. In addition, somatostatin receptor PET/CT and targeted histopathological assessmentare essential for tumour localisation, staging, and diagnostic confirmation. Finally, in selected inoperable patients with advanced carcinoid-related TR, heterotopic bicaval valve implantation may provide symptomatic relief by reducing caval backflow without directly manipulating the severely diseased tricuspid valve.

## Figures and Tables

**Table 1:** Main echocardiographic parameters before and after heterotopic bicaval valve implantation.

Parameter	Pre-Procedural TTE (April 2025)	Post-Procedural TTE (August 2025)
**Left ventricular parameters**		
LVEDVi, mL/m^2^	81	79
LVESVi, mL/m^2^	46	35
LVEF, %	44	55
LV GLS, % (absolute value)	16.0	17.5
LA volume index, mL/m^2^	49	52
**Right ventricular parameters**		
RV basal diameter, mm	60	59
RV mid diameter, mm	45	45
TAPSE, mm	20	17
RV FAC, %	34	54
RV S′, cm/s	15	23
RV free-wall strain, % (absolute value)	18	20
RA volume index, mL/m^2^	70	60
**Valvular findings**		
Mitral valve	Severe MR; mild MS	Moderate MR; mild MS
Aortic valve	Moderate AR	Moderate AR
Tricuspid valve	Torrential TR	Torrential TR
Pulmonary valve	No significant dysfunction	Mild-to-moderate PR
**TricValve system**	Not applicable	IVC prosthesis with moderate central regurgitation and reduced hepatic vein reflux; SVC prosthesis with mild regurgitation

Abbreviations: AR, aortic regurgitation; FAC, fractional area change; GLS, global longitudinal strain; IVC, inferior vena cava; LA, left atrium; LV, left ventricle; LVEDVi, left ventricular end-diastolic volume index; LVEF, left ventricular ejection fraction; LVESVi, left ventricular end-systolic volume index; MR, mitral regurgitation; MS, mitral stenosis; PR, pulmonary regurgitation; RA, right atrium; RV, right ventricle; SVC, superior vena cava; TAPSE, tricuspid annular plane systolic excursion; TR, tricuspid regurgitation.

## Data Availability

The data presented in this case report are not publicly available due to ethical and privacy restrictions related to patient confidentiality. Relevant anonymised clinical data, imaging findings, and histopathological information may be made available from the corresponding author upon reasonable request, subject to institutional approval and compliance with applicable ethical and legal requirements.

## Supplementary Materials

The following supporting information can be downloaded at: https://www.mdpi.com/article/10.3390/diagnostics16121942/s1. Video S1: Transthoracic echocardiography; Video S2: Transoesophageal echocardiography (transgastric view); Video S3: Agitated-saline infusion during transoesophageal echocardiography.

## Abbreviations

The following abbreviations are used in this manuscript:

5-HIAA	5-hydroxyindoleacetic acid
AR	Aortic regurgitation
CAVI	Caval valve implantation
CHD	Carcinoid heart disease
CS	Carcinoid syndrome
CT	Computed tomography
FAC	Fractional area change
GLS	Global longitudinal strain
IVC	Inferior vena cava
LA	Left atrium
LV	Left ventricle
LVEDV	Left ventricular end-diastolic volume
LVEF	Left ventricular ejection fraction
LVESV	Left ventricular end-systolic volume
MR	Mitral regurgitation
MS	Mitral stenosis
MVA	Mitral valve area
NET	Neuroendocrine tumour
NT-proBNP	N-terminal pro-B-type natriuretic peptide
NYHA	New York Heart Association
PET	Positron emission tomography
PET/CT	Positron emission tomography/computed tomography
PFO	Patent Foramen Ovale
PR	Pulmonary regurgitation
RA	Right atrium
RV	Right ventricle
SVC	Superior vena cava
TAPSE	Tricuspid annular plane systolic excursion
TEE	Transoesophageal echocardiography
TR	Tricuspid regurgitation
TV	Tricuspid Valve
TRI-SCORE	Tricuspid Regurgitation Impact Score
TTE	Transthoracic echocardiography
